# Is the Behavioural Gender Gap Decreasing? Evidence from Food Consumption in Swiss Single-Person Households

**DOI:** 10.3390/foods13172838

**Published:** 2024-09-06

**Authors:** Daria Loginova, Stefan Mann

**Affiliations:** Socioeconomics, Agroscope, 8356 Ettenhausen, Switzerland; daria.loginova@agroscope.admin.ch

**Keywords:** food, consumption, gender gap, Switzerland

## Abstract

While Switzerland has made some progress over the past few decades in treating men and women more equally, this study intends to find out whether Swiss men and women’s food consumption patterns also converged between 1990 and 2017. After analysing 1.8 million observations of one-member households’ food baskets, we found that gender gaps are increasing significantly for 16 of 70 studied foods, decreasing significantly for another 16 of 70 studied foods and not changing significantly for more than half of the studied foods. On average, the gender gap in food consumption in Switzerland has increased over time. We conclude that behavioural differences between genders and culturally induced gender differences (e.g., unequal career chances) are largely unrelated.

## 1. Introduction

The Cambridge Dictionary describes a gender gap as ‘a difference between the way men and women are treated in society, or between what men and women do and achieve’. The food gender gap is a social indicator that helps to understand the power distribution in society, as well as the cultural, financial and other aspects of quality of life [1]. Women experience food discrimination in that they systematically obtain less food than is needed for a healthy life and reproduction [2,3,4,5]. Furthermore, women have always demonstrated different food consumption patterns from men [6,7]. What Ramstetter and Habersack [8] termed the behavioural gender gap has been demonstrated, inter alia, through strong evidence that women consume less meat [9] and alcohol [10] than men, but are more inclined towards buying organic food [11].

There is a huge body of research indicating how various social and economic factors influence the patterns of food consumption depending on the socioeconomic environment. Switzerland is a good example of a country in which—due to income levels being more than ten times higher than the global mean—ethical, psychological, social, quality and health restrictions and motivations [12] shape food choices more than budgetary restrictions. Therefore, any gender gaps in this country will hardly be due to accessibility, but rather due to different preferences and resulting behaviours. At a time when attempts to overcome gender inequality are intensifying in Switzerland, partly with success, it may be time to examine whether the behavioural gender gap is shrinking. The aim of our study is to focus on food variety and measure the food gender gap for the widest range of foods and the longest period of time possible at the level of society, with all its inequalities. We selected food consumption as evidence of a gender gap, because the food gender gap is not only a reflection of equal rights, but also an indicator of the health, nutrition, attitudes, development and culture of the eaters [2,3,5].

This study contributes not only to the debate on behavioural gender gaps in food consumption [1,10,11], but also to marketing studies on gender differences [13,14,15]. Systematically considering the differences in consumption behaviour increases companies’ abilities to reach gender-specific target groups [16,17]. Particularly in food marketing, gender-specific strategies often focus mainly on gender-specific communication instruments [18,19], neglecting the fact that targeting only one gender may be the most promising strategy.

## 2. Hypothesising the Behavioural Gender Gap in Switzerland

It is fair to say that the gender gap in Switzerland is larger than in most European countries. Swiss women were granted the right to vote between 1971 and 1991, after a century-long debate. Since 2006, Global Gender Gap Reports by the World Economic Forum have been evaluating gender gaps in 102–146 countries, including Switzerland, which ranked only 63rd (score: 0.7) in economic participation and opportunity, 102nd (score: 0.978) in education attainment and 115th (score: 0.964) in health and survival in 2023 [20]. On pay equality, FOGE & FSO [21] reported a 19 per cent difference between genders. Nevertheless, the gender gap has been narrowing over the past few decades, both internationally and in Switzerland. Worldwide, Parro [22] reported that the education gender gap has narrowed between 1975 and 2005. Similar progress has been reported in terms of earnings [23] and labour market participation [24], and modest progress has been observed in Switzerland, which in total scored 0.7 in 2006 and 0.78 in 2023, respectively, in the World Economic Forum’s gender gap monitoring project [20]. Furthermore, Switzerland’s share of women in top career positions moderately increased, from 29 per cent in 1991 to 37 per cent in 2019 [25].

A large part of the literature on attitudinal and behavioural gender differences has assumed that these differences are induced culturally [26,27,28], but such gender gaps are often based on normative notions A widespread notion that women are inferior to men, at least in certain aspects [29], influences women’s self-perceptions and eventually their attitudes and behaviour. Imhof et al. [30] found that this type of gender gap has started to decrease, whereas Kost et al. [31] reported the opposite. In other fields, studies on the development of attitudinal and behavioural gender gaps have been inconclusive, be it on attitudes about foreign policy [32] or religiosity [33].

Food consumption in Switzerland is a convenient field from which to collect evidence on the development of the gender gap, not only because Switzerland has seen progress in closing the gender gap on a societal level, but also because major differences in eating patterns between men and women have been observed, as summarised in Table 1.

Most scholars have asserted that differences in food consumption are induced socially. If ‘real men don’t eat quiche’, as Gal and Wilkie [45] put it, and if beer advertising is directed mostly at males [46], similar messages may shape eating patterns according to which both men and women make their food choices. Under such circumstances, steps towards gender equality in society would be likely to soften gendered food stereotypes—shifts to which consumers would be likely to adapt. The gender similarities hypothesis confines natural differences between genders to very few points (not covering food consumption preferences [47]); therefore, it would be reasonable to hypothesise that the gender gap in food consumption has decreased in Switzerland between 1990 and 2020. However, a minority of social scientists have pursued what Saguy et al. [48] call a biological-essentialist view on gender differences, asserting that biological differences are the main drivers of behavioural differences between genders [49,50]. From such a perspective, behavioural differences in eating patterns are not likely to be neutralised through societal changes. One could argue that successfully creating more such equal opportunities and living conditions between genders might enhance female strengths in this regard. The less male dominance that exists in a society, the less pressure women will feel to adapt to male stereotypes and the more women can cherish genuine female food consumption patterns, which are not influenced by societal and other external factors (see Appendix A).

## 3. Materials and Methods

This paper investigates the gender gap and changes in food consumption during the 1990–2017 period in Switzerland. We aimed to discover differences in food consumption between single women and single men, and to quantify the dynamics of the gender gap for a wide range of foods. We used data from the Swiss Federal Statistics Office, which asked members of randomly chosen households in Switzerland to provide personal characteristics and, among other details, the volumes and costs of foods consumed in the household. We only selected households with one member because ‘social eating’ may affect patterns with multiple members present [37].

We calculated the consumption of each food type in each household and excluded the 0.5% of participants with the highest and 0.5% with the lowest consumption per person in the groups by food and year. After these eliminations, we used 1.8 million observations from 17,932 participants—7044 males (39%) and 10,888 females (61%)—concerning 70 aggregated foods (1780 combinations of disaggregated foods and years) in some of the several available years, namely 1990 and 2000–2017. We calculated average personal consumption in groups through a combination of disaggregated foods, years and gender.

To quantify trends in the dietary gender gap, we used a simple linear regression with robust estimates. The foods i are indexed as $i_{d}$ if they were disaggregated over time and $i_{a}$ if they were aggregated to consistent classifications over time. For foods $i\in \{i_{d},i_{a}\}$ and years t, we denoted average consumption (in kg or litres) for single men as $c_{i,M,t}$ and for single women as $c_{i,F,t}$, as well as a sum of consumption for all participants as $c_{i,t}$. Similarly, $e_{i,t}$ stands for total expenditure on food in both types of single-person household (in Swiss francs). We denoted the groups of foods using dummy variables $d_{g},g\in \{‘processed’,‘animal’,’liquid’\}$, in which $d_{i_{d},g}$ equals 1 if the food belongs to the category g and otherwise equals zero. The purchase price $p_{i,t}$ is calculated as in (1), while gender gaps $y_{i,t}$ and ${\tilde{y}}_{i,t}$ are calculated as in (2) and (3), respectively. We obtained estimates of gender gap correlations using regressions (4) and (5):

(1) $$p_{i_{d},t}=e_{i_{d},t}/c_{i_{d},t}$$

(2) $$y_{i,t}=c_{i,F,t}-c_{i,M,t}$$

(3) $${\tilde{y}}_{i,t}={|c}_{i,F,t}-c_{i,M,t}|,i=i_{d}$$

(4) $$y_{i_{a},t}=\alpha +\beta_{0,i_{a}}t+\varepsilon_{i_{a},t}$$

(5) $${\tilde{y}}_{i_{d},t}=\tilde{\alpha }+\beta_{0}t+\beta_{1}d_{i_{d},processed}+\beta_{2}d_{i_{d},animal}+\beta_{3}d_{i_{d},liquid}+\beta_{4}p_{i_{d},t}+{\tilde{\varepsilon }}_{i_{d},t}$$

in which α and $\tilde{\alpha }$ are constants, $\varepsilon_{i,t}$ and ${\tilde{\varepsilon }}_{i,t}$ are the error terms, and $\beta_{0,i}$ and $\beta_{j}$ denote the correlations of interest. The magnitude and significance of the estimates of $\beta_{0,i}$ and $\beta_{j}$ (i.e.$,{\hat{\beta }}_{0,i}$ and ${\hat{\beta }}_{j}$) are the present study’s focus. Following the design of Equation (5), Table 2 shows the descriptive statistics of the variables.

In our final model, 39% of foods were animal-based foods, 16% of foods were liquid and 49% of foods were processed. The biggest absolute gender gap was observed for mineral water in 2009 when men consumed 3.93 L more than women. No gender gap was observed for wild and rabbit meat in 2006.

## 4. Results

### 4.1. Foods by Gender Gap

The consumption of most foods increased, decreased or did not change with both genders, although the gender gap dynamics with foods were diverse (Table 3).

The gender gap increased in the purchases of baby food, beans and peas, cabbage vegetables, canned fruit, canned vegetables and mushrooms, cereal products, confectionery, fruits, kitchen herbs, mixed milk-based products, onions and garlic, pastry, pears and quinces, ready meals and soups, in which women’s consumption increased faster than men’s. The gender gap decreased for aroma and taste essences, canned fish, citrus (except lemons), pasta and poultry, in which men’s consumption increased faster than women’s, and for butter, margarine, potatoes, root vegetables, sugar and vegetables (stem and fruit), in which men’s consumption decreased slower than women’s.

In exceptional cases, robust estimates of trends were insignificant, but the resulting gender gap changed significantly, e.g., the gender gap decreased for bread, dried fruits and sausages and increased for grapes. The following 7 of 70 studied foods had no similarity in consumption dynamics across genders, but the gender gap did not increase: mineral water; tea and herbs; ham and bacon; cream; leafy vegetables; stone fruit; and spirits and liqueurs.

### 4.2. The Food Gender Gap

The gender gap is higher for liquids and lower for animal and processed foods (Table 4). The constant and trend coefficients and their significance allow for concluding that the average gap is significant and increasing over time. Our results also indicate that gender gaps in food consumption significantly correlate with prices.

The studied relations require more investigation in the future because the coefficient of determination in the current model is only 0.12, indicating that other food properties may likely increase the model’s explanatory power. However, testing the model’s significance indicates that the model is better than addressing the food gender gap with only a constant, which is why we conclude that the model contributes to knowledge about gender gaps in Switzerland.

### 4.3. Limitations

This study’s first limitation is the impossibility of quantifying the change and distribution of consumption across genders within households with more than one member. The second limitation entails territorial and time coverage. We tested the hypothesis for Swiss data during a period from 1990 to 2017. Finally, many factors lay beyond our study’s scope because we only aimed to quantify the gender gap, no matter how impactful the other factors were.

## 5. Discussion

Our results suggest that there are gender differences (gaps) in the food consumption of single-person households, and these gaps do not decrease on average, but significantly change for particular foods. This paper employed 1.8 million observations that Swiss residents collected accurately over the period from 1990 to 2017 and demonstrated that in Switzerland (1) the gender gap in food consumption exists for selected foods; (2) gender gaps are increasing significantly for 16 of 70 studied foods, decreasing significantly for 16 of 70 studied foods and not changing significantly for more than half of the studied foods; (3) the consumption of 31 of 70 studied foods increased for both genders and decreased for 10 foods among both genders; (4) the genders demonstrated diverse trends (by the signs of the trends) only with seven exceptional foods from the 70 studied (mineral water, tea and herbs, ham and bacon, cream, leafy vegetables, stone fruit, and spirits and liqueurs); and (5) on average, the gender gap in food consumption in Switzerland increased over time. Our results allow us to reject the hypothesis about a shrinking behavioural gender gap in Switzerland. Rather, it can be demonstrated that the gender gap in consumption behaviour among single-person households increased in the years studied.

Previous studies have demonstrated that gender matters for sustainable consumption [51] and that women tend to consume more green foods and pollute less compared with men [7,52]. The dietary behaviour that we discovered in our paper is specific to Switzerland, and it is certainly worthwhile to explore if it also applies to other countries. Our study is the first to examine the gender gap for various foods at the level of society, with its inequalities, and observing it over a long period.

## 6. Conclusions

While the trend that we found certainly should not be overestimated, one should allow for the possibility that women may play a key role in developing sustainable consumption patterns and finally share them with men. In this case, a ‘difference approach’ to genders should not be seen as a tool to keep women oppressed, but rather as a way to help liberate women from societal expectations shaped by men, including when it comes to the food on their tables. This idea builds on the empirical evidence on gender gaps that researchers have examined for many other spheres of life, such as academic careers, education choices, voting, food security and the consumption of specific food items.

The results should also be able to support the future development of food marketing strategies. Particularly for the 16 product groups in which gender gaps are increasing, it will be worthwhile to consider marketing strategies that are targeted at just one gender, or to develop different strategies for men and women. Marketing scholars have already shown how a consolidated understanding of the existence and development of gender gaps in food consumption can be used for appropriate marketing strategies.

Future studies in the academic realm should be more cautious when it comes to the level of product aggregation and consider the level and dynamics of gender differences. One of possible avenues for further research is studying the causalities of widening and narrowing food gender gaps. In this respect, our paper serves only as a starting point for a refined analysis.

## Figures and Tables

**Table 1:** Literature on food consumption across genders in Switzerland.

Author (Year)	Method (Period)	Result (Territory Restriction)
Loginova and Mann (2024) [34]	Trend analysis and pattern classification (1990–2020) °°°	‘Single men consume more drinks, canned and prepared fish and meat, sausages and pasta compared to single women that have higher consumption of the rest of gender-dependent foods’.
Tschanz et al. (2022) [35]	Multivariable linear regression. (2014/2015) °°	The male population consumed almost twice as much meat as women, which was observed for all meat types except white meat.
Baur et al. (2022) [36]	Swiss-specific Nutritional Index (Aug. 2018–Sep. 2018) °	Males demonstrated higher consumptions of processed meat, red meat, alcoholic beverages and sodas, and lower intakes of whole grains, vegetables and fruits. Females had lower carbon footprints and ate substantially healthier than males.
Inanir et al. (2020) [37]	Multivariable linear regression (Jan. 2014–Feb. 2015) °°	Energy-standardised dairy intake was 20.7 g/1000 kcal higher for women than men.
Steinbach et al. (2020) [38]	Logistic regression (Jan. 2014–Feb. 2015) °°	Women were less likely to be frequent meat eaters.
Wozniak et al. (2020) [39]	Questionnaire and logistic regression (2005–2017) °°°	Pescatarians and flexitarians were more likely to be women (Geneva).
Schneid Schuh et al. (2018) [40]	Logistic regression (between 1993 and 2016) °°°	Compliance with Swiss dietary guidelines increased for combinations: fruits—men; vegetables—both genders; meat—women; dairy products—both genders younger than 64 years old (Geneva).
Chatelan et al. (2017) [41]	Differences in food consumption and compliance to the Swiss food-based dietary guidelines (2014–2015) °°	‘Daily energy intakes for men and women (i.e., 2538 and 1899 kcal) were close to recommendations for moderately active adults’.
Mann et al. (2012) [42]	Conjoint and regression analyses (Autumn, 2009) °	‘Urban women appear to be considerably more attracted to organic wine than rural men’.
Galobardes et al. (2001) [43]	Multiple linear regression (1993–1998) °°°	Women consumed smaller amounts of pasta and potatoes than men. (Geneva).
Eichholzer and Bisig (2000) [44]	Bivariate analyses and multivariate logistic regressions (1992/1993) °°°	Men were found to consume daily meat or meat products more frequently. A preference for red meat rather than white meat was observed more often in men.

° Less than 1000 participants; °° 2057 participants (Swiss National Nutrition Survey menuCH); °°° over 5000 participants.

**Table 2:** Descriptive statistics.

Variable	Minimum and Maximum	Average
Absolute gender gap	0.00 and 3.93	0.16
Time trend	0 (in 1990) and 27 (in 2017)	18
Processed	0 and 1	0.49
Animal	0 and 1	0.39
Liquid	0 and 1	0.16
Price	0.63 and 64.6	13.4

**Table 3:** Trends in consumption and in dietary gender gap, by food.

Consumption for Both Genders	Gender Gap (Women’s Consumption Minus Men’s Consumption)
	**Increasing**:
increases	Baby food; beans and peas; cabbage vegetables; canned fruit; canned vegetables and mushrooms; cereal products; confectionery; fruits; kitchen herbs; mixed milk-based products; onions and garlic; pastry; pears and quinces; ready meals; soups
decreases	-
does not change	Grapes
	**Decreasing**:
increases	Aroma and taste essences; canned fish; citrus (except lemons); pasta; poultry
decreases	Butter; margarine; potatoes; root vegetables; sugar; vegetables (stem and fruit)
does not change	Bread; dried fruit; sausages
	**Does not change**
increases	Bananas; berries; cocoa and chocolate; dried vegetables and mushrooms; fish; fish and seafood prepared; lemons; olive oil; seafood; syrups; vegetarian soy products
decreases	Apples; coffee and substitutes; ice cream; tomatoes
does not change	Beef; beer; canned milk and milk powder; cheese and curd; egg; flours; honey; jam; milk; non-alcohol drinks; nuts; oils and fats (except olive oil); other meat; pork; rice; veal; wines; yoghurt; fresh mushrooms; canned meat; wild and rabbit meat; sheep meat and goat meat; horse meat

Exceptional foods: mineral water; tea and herbs; ham and bacon; cream; leafy vegetables; stone fruit; spirits and liqueurs.

**Table 4:** Results from a model explaining the dietary gender gap.

Explanatory Variable	$\mathrm{Estimate}\;{\hat{\beta }}_{j}$ (Standard Error)
Intercept	0.166 (0.03) ***
Time trend	0.006 (0.001) ***
Food properties:	
Processed	−0.05 (0.02) *
Animal	−0.05 (0.02) **
Liquid	0.17 (0.02) ***
Price	−0.007 (0.0008) ***

These are results of assessed Equation (5). Significance codes: ‘***’ = *p* ≤ 0.001; ‘**’ = *p* ≤ 0.01; ‘*’ = *p* ≤ 0.05; Years under study: 1990, 2000–2017. Number of observations: 1631. The model is significant according to the F-test on overall significance in regression analysis, in which H0: the fits of the intercept-only model and assessed model are equal (the model is significant if H0 is rejected). The coefficient of determination is 0.12.

## Data Availability

The original contributions presented in the study are included in the article, further inquiries can be directed to the corresponding author.

## Appendix A

**Table A1:** Consumption and gender gap trends.

Food	Men	Women	Gender Gap
Apples	−0.035 (0.004) ***	−0.049 (0.011) **	−0.014 (0.008) .
Aroma and taste essences	0.024 (0.002) ***	0.018 (0.002) ***	−0.006 (0.001) ***
Baby food	0.031 (0.004) ***	0.041 (0.006) ***	0.01 (0.004) *
Bananas	0.027 (0.009) **	0.037 (0.013) *	0.01 (0.005) .
Beans and peas	0.031 (0.006) ***	0.052 (0.01) ***	0.021 (0.005) **
Beef	0.008 (0.007)	0.006 (0.008)	−0.002 (0.002)
Beer	0.092 (0.046) .	0.022 (0.012) .	−0.07 (0.035) .
Berries	0.01 (0.003) **	0.009 (0.004) *	−0.001 (0.002)
Bread	0.059 (0.049)	0.039 (0.045)	−0.02 (0.005) **
Butter	−0.008 (0.001) ***	−0.011 (0.002) ***	−0.003 (0.001) *
Cabbage vegetables	0.037 (0.005) ***	0.047 (0.006) ***	0.01 (0.002) **
Canned fish	0.201 (0.036) ***	0.155 (0.033) ***	−0.046 (0.009) ***
Canned fruit	0.044 (0.007) ***	0.062 (0.012) ***	0.018 (0.005) **
Canned milk and milk powder	0 (0.001)	−0.001 (0.001)	−0.001 (0)
Canned vegetables and mushrooms	0.049 (0.012) **	0.083 (0.018) ***	0.035 (0.007) ***
Cereal products	0.036 (0.004) ***	0.042 (0.005) ***	0.006 (0.002) **
Cheese and curd	0.014 (0.019)	0.02 (0.022)	0.005 (0.003)
Citrus (except lemons)	0.029 (0.008) **	0.021 (0.009) *	−0.008 (0.002) ***
Cocoa and chocolate	0.018 (0.003) ***	0.019 (0.003) ***	0.001 (0.001)
Coffee and substitutes	−0.004 (0) ***	−0.005 (0.001) ***	−0.001 (0.001) .
Confectionery	0.017 (0.003) ***	0.02 (0.003) ***	0.004 (0.001) *
Cream	−0.006 (0.002) **	−0.008 (0.004) .	−0.002 (0.003)
Dried fruits	0.001 (0.001)	−0.001 (0.001)	−0.002 (0.001) **
Dried vegetables and mushrooms	0.026 (0.005) ***	0.028 (0.005) ***	0.002 (0.001) .
Egg	−0.002 (0.003)	−0.004 (0.005)	−0.002 (0.001)
Fish	0.005 (0.001) ***	0.004 (0.001) ***	−0.001 (0.001)
Fish and seafood prepared	0.002 (0) ***	0.002 (0) ***	0 (0) .
Flours	0.001 (0.001)	0 (0.001)	0 (0.001)
Fruits	0.009 (0.001) ***	0.013 (0.001) ***	0.004 (0.001) ***
Grapes	−0.002 (0.002)	0.004 (0.002)	0.006 (0.002) **
Ham and bacon	0.008 (0.004) *	0.007 (0.004)	−0.001 (0.001)
Honey	0.015 (0.008) .	0.015 (0.011)	0 (0.003)
Ice cream	−0.014 (0.001) ***	−0.014 (0.001) ***	0 (0)
Jam	−0.003 (0.002)	−0.003 (0.002)	0 (0)
Kitchen herbs	0.02 (0.003) ***	0.031 (0.005) ***	0.011 (0.002) ***
Leafy vegetables	−0.006 (0.001) ***	0 (0.004)	0.006 (0.004)
Lemons	0.064 (0.013) ***	0.07 (0.014) ***	0.006 (0.005)
Margarine	−0.004 (0) ***	−0.007 (0.001) ***	−0.003 (0.001) ***
Milk	−0.022 (0.04)	−0.016 (0.043)	0.006 (0.006)
Mineral water	0.102 (0.031) **	−0.04 (0.012) **	−0.142 (0.036) **
Mixed milk-based products	0.022 (0.003) ***	0.03 (0.003) ***	0.008 (0.002) **
Non-alcoholic drinks	−0.163 (0.153)	−0.141 (0.133)	0.022 (0.021)
Nuts	−0.001 (0.001)	0 (0.001)	0.001 (0.001)
Oils and fats (except olive oil)	0.003 (0.002)	0.005 (0.003)	0.002 (0.001)
Olive oil	0.004 (0.001) **	0.006 (0.001) ***	0.003 (0.001) .
Onions and Garlic	0.015 (0.003) ***	0.027 (0.006) ***	0.012 (0.002) ***
Other meat	0.003 (0.002) .	0.002 (0.001) .	−0.001 (0)
Pasta	0.02 (0.005) ***	0.017 (0.004) **	−0.004 (0.001) **
Pastry	0.13 (0.01) ***	0.141 (0.011) ***	0.011 (0.003) *
Pears and quinces	0.036 (0.009) **	0.055 (0.014) **	0.018 (0.005) **
Pork	0.009 (0.007)	0.006 (0.005)	−0.003 (0.002) .
Potatoes	−0.021 (0.008) *	−0.04 (0.013) **	−0.019 (0.005) **
Poultry	0.01 (0.002) ***	0.008 (0.002) **	−0.003 (0.001) **
Ready meals	0.001 (0) ***	0.004 (0) ***	0.003 (0) ***
Rice	0.003 (0.002)	0 (0.001)	−0.003 (0.002)
Root vegetables	−0.013 (0.002) ***	−0.022 (0.004) ***	−0.009 (0.003) **
Sausages	0.021 (0.015)	0.012 (0.011)	−0.008 (0.004) *
Seafood	0.002 (0.001) **	0.002 (0) ***	0 (0.001)
Soups	0.001 (0) ***	0.001 (0) ***	0.001 (0) **
Spirits and liqueurs	0.001 (0.002)	0.003 (0.001) *	0.002 (0.001)
Stone fruit	−0.008 (0.002) **	−0.002 (0.007)	0.006 (0.005)
Sugar	−0.005 (0.002) *	−0.014 (0.004) **	−0.01 (0.002) **
Syrups	0.14 (0.031) ***	0.12 (0.027) ***	−0.019 (0.011)
Tea and herbs	0 (0)	−0.002 (0) ***	−0.001 (0) **
Tomatoes	−0.017 (0.002) ***	−0.02 (0.002) ***	−0.003 (0.002)
Veal	0.001 (0.001)	0 (0.002)	−0.001 (0.001)
Vegetables (stem and fruit)	−0.007 (0.003) *	−0.02 (0.007) *	−0.013 (0.004) **
Vegetarian soy products	0.008 (0.001) ***	0.007 (0.001) ***	−0.001 (0.001)
Wines	0.065 (0.049)	0.072 (0.037) .	0.007 (0.014)
Yoghurt	−0.012 (0.015)	−0.011 (0.019)	0.001 (0.005)

Significance codes: ‘***’ = *p* ≤ 0.001; ‘**’ = *p* ≤ 0.01; ‘*’ = *p* ≤ 0.05; ‘.’ = *p* ≤ 0.1. Zero insignificant trends: fresh mushrooms; canned meat; wild and rabbit meat; sheep meat and goat meat; horse meat.
